# Bacterioplankton communities reveal horizontal and vertical influence of an Island Mass Effect

**DOI:** 10.1111/1462-2920.16092

**Published:** 2022-06-12

**Authors:** Jacqueline Comstock, Craig E. Nelson, Anna James, Emma Wear, Nicholas Baetge, Kristina Remple, Amethyst Juknavorian, Craig A. Carlson

**Affiliations:** ^1^ Department of Ecology, Evolution and Marine Biology and Marine Science Institute University of California Santa Barbara Santa Barbara CA USA; ^2^ Daniel K. Inouye Center for Microbial Oceanography: Research and Education, Department of Oceanography and Sea Grant College Program University of Hawai'i at Mānoa Honolulu HI USA; ^3^ Pasadena Unified School District Pasadena CA USA

## Abstract

Coral reefs are highly productive ecosystems with distinct biogeochemistry and biology nestled within unproductive oligotrophic gyres. Coral reef islands have often been associated with a nearshore enhancement in phytoplankton, a phenomenon known as the Island Mass Effect (IME). Despite being documented more than 60 years ago, much remains unknown about the extent and drivers of IMEs. Here we utilized 16S rRNA gene metabarcoding as a biological tracer to elucidate horizontal and vertical influence of an IME around the islands of Mo′orea and Tahiti, French Polynesia. We show that those nearshore oceanic stations with elevated chlorophyll *a* included bacterioplankton found in high abundance in the reef environment, suggesting advection of reef water is the source of altered nearshore biogeochemistry. We also observed communities in the nearshore deep chlorophyll maximum (DCM) with enhanced abundances of upper euphotic bacterioplankton that correlated with intrusions of low‐density, O_2_ rich water, suggesting island influence extends into the DCM.

## Introduction

Phytoplankton production provides a fundamental source of energy at the base of marine food webs (Duarte & Cebrián, 1996), where the distribution of phytoplankton biomass dictates the trophic structure of marine ecosystems and the production of the world's fisheries (Chassot *et al*., 2010; Iverson, 1990). In oligotrophic waters, the ecological impacts of increased phytoplankton production are especially pronounced, with oceanic waters around coral reef islands and atolls displaying significantly enhanced primary production, elevated chlorophyll *a* (Chl), elevated animal biomass and unique biogeochemistry (Andrade *et al*., 2014; Dandonneau & Charpyt, 1985; Doty & Oguri, 1956; Gove *et al*., 2016; James *et al*., 2020; Jones, 1962; Martinez & Maamaatuaiahutapu, 2004; Palacios, 2002; Raapoto *et al*., 2019; Signorini *et al*., 1999; Vollbrecht *et al*., 2021). This observed increase in phytoplankton biomass around reef island and atoll ecosystems – the ‘Island Mass Effect’ (IME) – was first observed more than 60 years ago and has been attributed to an increase in bioavailable nutrients in the euphotic zone surrounding the islands and atolls (Doty & Oguri, 1956). IMEs have been observed to be a common feature throughout the tropical Pacific (Gove *et al*., 2016), yet much remains unknown about drivers of this phenomenon, the depth to which enhanced biological activity penetrates the water column, or its impact on microbial assemblages and metabolic potentials.

An IME has recently been documented surrounding the islands of Mo′orea and Tahiti (James *et al*., 2020). Located in the South Pacific Subtropical Gyre, the island of Mo′orea, French Polynesia, is surrounded by some of the least biologically productive waters in the global ocean (Longhurst *et al*., 1995). However, the backreef – the shallow lagoon behind barrier reefs – of this coral reef system displays unique chemical and biological signatures such as elevated nitrate and Chl, and depleted dissolved organic carbon (DOC) concentrations when compared to the open waters surrounding the island (Nelson *et al*., 2011; Leichter *et al*., 2013). Transport and retention of key biogeochemical constituents such as inorganic nutrients and organic particles within tropical reefs are thought to support the high biomass and increased productivity observed (Johannes *et al*., 1972; Odum & Odum, 1995). James *et al*. (2020) showed that the islands of Mo′orea and Tahiti have the potential to alter the biological activity and biogeochemistry of oceanic water in their vicinity, documenting enhanced levels of net primary productivity (NPP), Chl, heterotrophic bacterioplankton productivity (BP) and particulate organic carbon (POC) in oceanic waters within 15 km of the islands relative to farther offshore.

The horizontal and vertical influence of the observed IME around Mo′orea and Tahiti has not previously been investigated. Mechanisms contributing to observed IMEs have been diverse and found to vary by location. These include nutrient and organic matter inputs from terrestrial sources, such as riverine or submarine groundwater discharge (Anderson *et al*., 2002; Dandonneau & Charpyt, 1985; Garrison *et al*., 2003; Knee *et al*., 2016; Street *et al*., 2008). Anthropogenic factors such as land‐use change, urban development and wastewater discharge can also enhance nutrient inputs into nearshore waters (Anderson *et al*., 2002; Dandonneau & Charpyt, 1985; Knee *et al*., 2016; Street *et al*., 2008). Beyond direct terrestrial inputs, coral reef organisms have been found to biogeochemically alter reef water (Atkinson, 1987; Falter *et al*., 2004; Haas *et al*., 2011, 2013; Wyatt *et al*., 2010). Additionally, physical processes can impact movement and retention of nutrients around islands. Interaction of currents with island bathymetry may form eddies and induce upwelling (Hamner & Hauri, 1981; Takahashi *et al*., 1981; Wolanski & Hamner, 1988), and internal waves can interact with bathymetry, creating instabilities and delivering deeper waters with distinct biogeochemical and physical properties to the surface (Carter *et al*., 2006; Leichter *et al*., 2003, 2012; Wyatt *et al*., 2020). Around Mo′orea, a counterclockwise flow has been observed and has been suggested as a mechanism for physical retention of biogeochemically altered reef water around the island (James *et al*., 2020; Leichter *et al*., 2013). This counterclockwise circular flow is thought to circulate advected reef water in the oceanic environment around the island, keeping this biogeochemically altered water near Mo′orea and allowing for potential re‐entrainment into the reef system (Leichter *et al*., 2013).

Here we provide a comprehensive synoptic survey of 16S rRNA gene metabarcoding data collected around the islands of Mo′orea and Tahiti in July 2014 in conjunction with the physical and biogeochemical parameters described in James *et al*. (2020). We describe the distribution of bacterioplankton communities as a potential biological tracer to investigate whether the biological variability associated with this IME could be due to advection of backreef water or upwelling from depth. We also use these phylogenetic markers to assess how an IME may affect the microbial composition of the deep chlorophyll maximum (DCM) and upper mesopelagic.

## Methods

### Site description

This study was carried out within a grid (51 km × 67 km) around the Society Island of Mo′orea. Mo′orea is a 1.5–2‐million‐year‐old (Neall & Trewick, 2008) high volcanic island surrounded by barrier reefs within 0.5–1 km of shore. There are fringing reefs bordering the island (~10 m deep) and a shallow (<3 m) backreef lagoon with a mixture of coral, algae and barren sands. The backreef lagoons connect to open ocean by reef pass channels that occur at approximately 5–10 km intervals around the island. These reef pass channels often correspond with embayments of varying sizes. The forereef has a high coral density and drops steeply to depths exceeding 500 m within 1 km offshore from the reef crest (Nelson *et al*., 2011; James *et al*., 2020). Waves drive water from the forereef across the reef crest into the backreef lagoon (Hench *et al*., 2008). Water then flows parallel to the island into the reef pass channels and is advected offshore through the passes. Movement within the reef environment is rapid (averaging 0.2 m s^−1^ with limited tidal influence; Mo′orea is near a local M2 amphidromic point) and maintains a short residence time of hours to days within the backreef environment (Hench *et al*., 2008; Knee *et al*., 2016). Additionally, a weak counterclockwise flow has been observed around Mo′orea, and has been suggested to be a mechanism by which advected reef water can stay near the island (Leichter *et al*., 2013).

### Sampling scheme

**Fig. 1:**
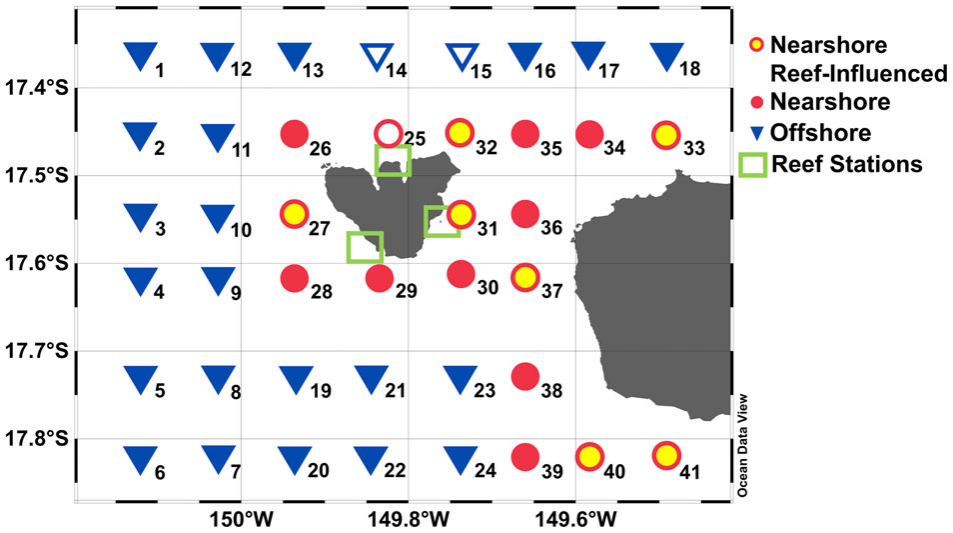
Map of study area around Mo'orea and Tahiti and shipboard sampling grid during July/August 2014. Symbol outline colours refer to spatial classifications following James *et al*. (2020): Offshore stations (blue) and nearshore stations (red). Nearshore stations exhibiting influence of reef bacterioplankton are designated as ‘nearshore reef‐influenced’ (yellow interior). An additional 46 reef stations were sampled within the green rectangles (map in Supplementary Fig. 1). Offshore and nearshore stations with white interior represent stations with physical and biogeochemical measurements but no bacterioplankton community data.

Water samples were collected aboard the R/V *Kilo Moana* coincident with biogeochemical and physical oceanographic variables over the surface 500 m at 41 hydrographic stations in a grid (17.359°S–17.820°S by 149.491°W–150.121°W) around the Society Islands of Mo′orea and Tahiti in French Polynesia (Fig. 1) from July 27 to August 7, 2014. Stations occupied by the R/V *Kilo Moana* are referred to as ‘oceanic’ stations. Staying with the description in James *et al*. (2020), the oceanic stations were further categorized as ‘nearshore’ or ‘offshore’ if they were within or beyond 15 km of the islands respectively. Samples from the R/V *Kilo Moana* were collected nominally at 10, 25, 50, 75, 100, 150, 200, 250, 300 and 500 m by a conductivity, temperature, depth (CTD) profiling rosette affixed with 24 10 L Niskin bottles. We define all depths within the surface 75 m as being representative of the upper euphotic zone, depths between 90 and 125 m as the DCM and depths at 150 m and deeper as the upper mesopelagic.

During the same time period an additional 46 stations within the reef environment on the north, west and east sides of Mo′orea were also sampled from the surface to as deep as 25 m and are referred to as ‘reef’ stations. Reef stations were sampled with a horizontal Niskin bottle using a polyester rope and weighted messenger, with collections at two to three depths spanning the entire water column of the forereef, backreef and reef pass environments (nominally 1, 10 and 25 m in forereef habitats; in backreef habitats bottom samples were collected 20 cm above the benthos, typically at 2–3 m depth). Measured biogeochemical variables included dissolved inorganic nutrients (nitrate, nitrite, ammonium and phosphate), NPP, chlorophyll *a* (Chl), phaeopigments, heterotrophic BP, bacterial abundance, DOC, POC and particulate organic matter nitrogen isotopes (POM‐δ^15^ N). A detailed description of these data and standard operating procedures are available in James *et al*. (2020). Mixed layer depth (MLD) was calculated as the depth where density was 0.01 kg m^−3^ greater than the local near‐surface value. All CTD and biogeochemical data are available in the National Science Foundation (NSF) Environmental Data Initiative (EDI) data catalogue under the package ID knb‐lter‐mcr.5035.1.

### DNA collection and extraction

Two litres of whole seawater were gravity‐filtered onto a 0.22 μm pore‐size 47 mm polyethersulfone flat filter (Pall Supor®) housed in a polycarbonate filter cartridge that was attached directly to the Niskin bottle (nearshore and offshore samples) or after transfer to shore in a 5 L HDPE carboy held in a chilled cooler (reef samples). All filters were stored dry at −40°C until further processing. DNA was extracted using the Qiagen DNEasy Blood & Tissue Kit using manufacturer instructions modified as follows: 1 ml of sucrose lysis buffer (40 mmol L^−1^ EDTA, 50 mmol L^−1^ Tris HCl, 750 mmol L^−1^ sucrose, 400 mmol L^−1^ NaCl, pH adjusted to 8.0), 100 μl of sodium dodecyl sulfate (10% wt./vol.) and 10 μl of 20 mg ml^−1^ proteinase K were added, samples were incubated at 55°C for 2 h, then refrozen until extraction. Additionally, to increase DNA yield, 750 μl of lysate mixture was extracted by running three 250 μl volumes through the same microcentrifuge column with 750 μl of Qiagen AW1 buffer. Extracted genomic DNA was stored at −40°C until amplification. Samples from three stations (oceanic stations 14, 15 and 25; Fig. 1) were compromised prior to extraction and were removed from further analysis.

### Amplicon library sequencing and bioinformatics

Amplification of the V4 region of the 16S rRNA gene was performed using the 515F‐Y (5′‐GTGYCAGCMGCCGCGGTAA‐3′) and 806RB (5′‐GGACTACNVGGGTWTCTAAT‐3′) primers (Apprill *et al*., 2015; Parada *et al*., 2016). PCR‐grade water process blanks and mock communities (BEI Resources mock communities HM‐782D and HM‐783D) were included with each 96‐well plate of samples as quality control checks. Amplicons were cleaned and normalized using SequalPrep plates (Invitrogen), pooled at equal volumes, concentrated using Amicon Ultra 0.5 ml centrifugal tubes (Millipore), gel extracted using the QIAquick Gel Extraction Kit to remove non‐target DNA (Qiagen) and sequenced on an Illumina MiSeq using PE250 chemistry at University of California (UC), Davis DNA Technologies Core.

Samples were demultiplexed at UC Davis DNA Technologies Core. Fastq files were quality filtered and merged using the dada2 package (version 1.13) in R (Callahan *et al*., 2016). Chimaeras were removed *de novo* using the removeBimeraDenovo function in the dada2 package. Amplicon sequence variants (ASVs) were given a taxonomic assignment in the dada2 package using the assignTaxonomy command and the SILVA database (version 132) (Quast *et al*., 2013). After sequence classification, and initial assessment of plastid abundance, plastid sequences were removed and remaining reads were subsampled to 3000 reads per sample to standardize read depth. Samples below a read depth of 3000 were removed from further analysis which amounted to 19 out of 478 environmental samples. Mock communities and negative controls were checked to confirm consistency in amplification and lack of contamination between PCR plates and then removed from further analysis. All DNA sequence data are available in the National Center for Biotechnology Information (NCBI) Sequence Read Archive (SRA) under project number PRJNA773129

### Statistical analyses

For all multivariate analyses, ASV relative abundances were pre‐treated using an angular transformation to normalize the dataset. Bray–Curtis dissimilarity matrices and NMDS ordinations were calculated in R (v4.0) using the vegedist and metaMDS functions in vegan (v2.5) (Oksanen *et al*., 2020). NMDS ordinations were calculated and visualized on two axes, with stress values for all ordinations falling below 0.2 and reported with each NMDS ordination. Up to 20 iterations of NMDS ordination were performed until convergence. ASVs found in only one sample were removed when clustering DCM communities to better investigate the impact of isopycnal intrusions on DCM community, but were kept for all other analyses. A ‘horseshoe effect’ can be observed in NMDS ordinations with communities collected over several depths. The bending of dissimilar samples towards one another occurs when the analysis struggles with discriminating between samples that do not share common features (Morton *et al*., 2017). To avoid misinterpretation of overall trends due to this ‘horseshoe effect’, dendrograms were used to emphasize separation of bacterioplankton communities by depth. Dendrograms were created using Ward dissimilarity of Bray–Curtis dissimilarity matrices and were generated using JMP Pro 15. permutational multivariate analyses of variance (PERMANOVA) and multivariate homogeneity of group dispersion analyses were run using adonis, and betadisper functions in vegan (v2.5). Pairwise PERMANOVAs were run using pairwiseAdonis. *T*‐tests were run in R using the stats package (v3.6.2). One‐way ANOVAs were run in SAS using JMP Pro 15 (SAS Institute, Cary, NC). Analyses run can be found at https://github.com/jacquicomstock/KM14-16-16SrDNA.git.

## Results

### Overall dynamics: bacterioplankton communities separate by location and by depth

**Fig. 2:**
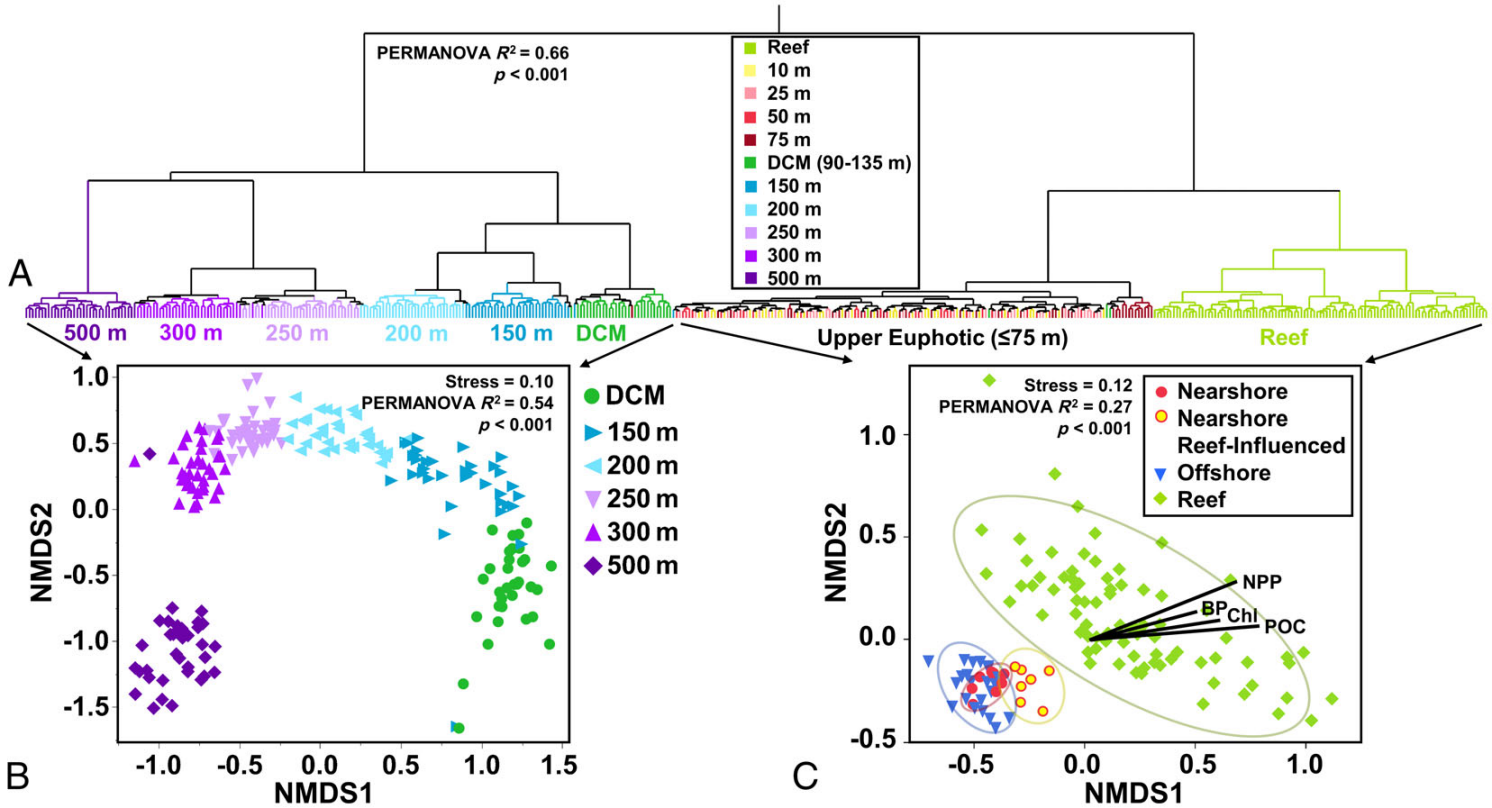
Community variation of bacterioplankton among sampling stations and depths. Hierarchical clustering of samples according to Bray–Curtis dissimilarity of community relative abundance profiles using Ward's minimum variance method show separation by depth and location (A). Non‐metric multidimensional scaling (NMDS) ordination of bacterioplankton communities show separation by depth in samples subset from the deeper depth strata – DCM through the mesopelagic (B) – and NDMS ordination of communities from the upper 10 m and shallow reef environments with overlaid environmental vectors of net primary production (NPP), heterotrophic bacterioplankton production (BP), particulate organic carbon (POC) and chlorophyll *a* (Chl) (C).

**Fig. 3:**
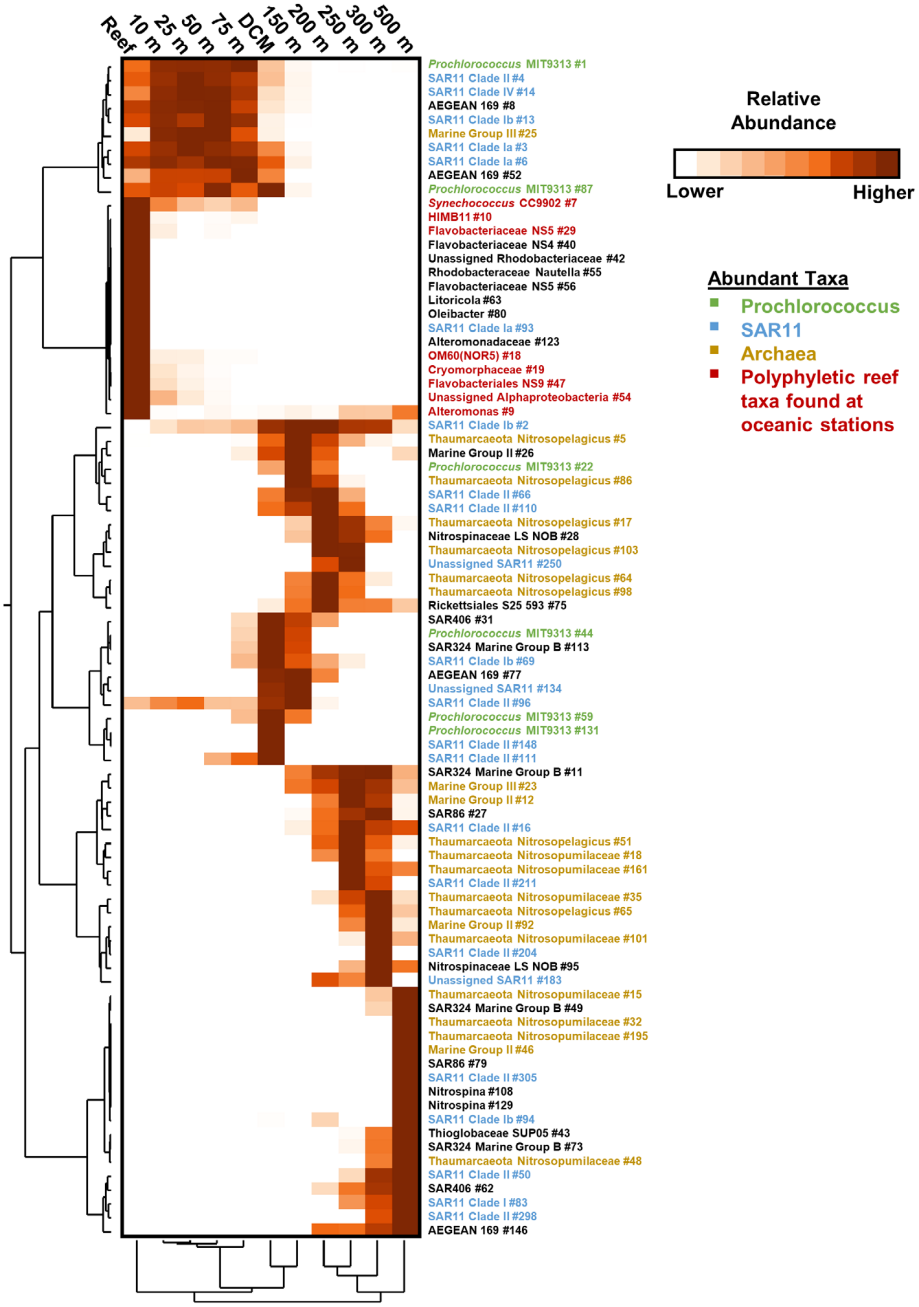
Depth enrichment stratification of abundant and widespread bacterioplankton ASVs. Heatmap of standardized mean relative abundance of bacterioplankton ASVs found in at least three samples with a relative abundance higher than 4%. ASVs and sampling locations are clustered using Ward's method as dendrograms at left and bottom respectively. Ranges of relative abundances that correspond to the standardized heatmap colouration for each bacterioplankton ASV are provided in Supplementary Fig. 7.

Overall, 459 unique environmental samples were successfully sequenced containing 2980 ASVs, with 2751 non‐singleton ASVs. Bacterioplankton communities clustered strongly by depth (PERMANOVA *R* ^2^ = 0.66, *p* < 0.001) with distinct community clusters at every sampled depth deeper than 75 m (Fig. 2A and B). Within the upper euphotic, samples from the upper 75 m for the oceanic stations clustered together, apart from a handful of 75 m samples that clustered separately (Fig. 2A). Reef stations (1–25 m) clustered separately from upper euphotic oceanic stations (Fig. 2C). Figure 3 shows the distribution of ASVs found in at least three samples with a relative abundance greater than 4%. All ASVs displayed variation in relative abundance by depth, with each depth below 75 m displaying enhanced abundance of a unique suite of ASVs. All depths within the upper 75 m showed similar relative abundances for the majority of ASVs resolved (Fig. 3). Additionally, reef stations displayed strong enhancement of ASVs largely absent or in low abundance at oceanic stations.

The whole 16S rRNA gene metabarcoding community contained prokaryotes and eukaryote plastids, with chloroplast plastid sequences averaging just 1.18% of all ASVs in the surface 75 m, 4.9% of all ASVs within the DCM (90–135), and 0.33% of all ASVs within the upper mesopelagic (150–500). However, ASVs associated with cyanobacterial lineages averaged 32% of all ASVs and 96.4% of the photoautotrophic ASVs in the upper euphotic. In the DCM, cyanobacteria averaged 15.9% of all ASVs and 76.4% of photoautotroph ASVs. These data indicate that the chlorophyll variability at both depth zones was likely primarily driven by bacterioplankton rather than eukaryotes. However, it is important to note that due to a lack of a mechanical lysis step during DNA extraction, plastid abundance may be underestimated. For all further analyses, plastid sequences were removed and samples were rarefied to standardize read depth and ensure even sampling effort.

Several ASVs that were associated with the *Prochlorococcus* clade stratified vertically. The most abundant cyanobacterium in the upper 75 m of the water column was the *Prochlorococcus* ASV #1 that averaged 31.1% and 5.7% of the total prokaryotic community in the upper 75 m and DCM respectively. The *Prochlorococcus* ASV (#44), the fourth most abundant of cyanobacteria in the dataset, was found primarily within the DCM with an average relative abundance of 3.5% and reached a relative abundance of 14.4% at one DCM station. A third deep *Prochlorococcus* ASV (#22) was found primarily at the base of the euphotic zone (i.e. ~150 m), averaging 2.3% of the community at that depth (Fig. 3).

### Reef bacteria found at nearshore oceanic stations with enhanced chlorophyll *a*

James *et al*. (2020) reported elevated Chl, NPP, POC and BP for nearshore stations relative to offshore stations. Chl distribution within the upper euphotic revealed elevated Chl concentrations at nearshore stations in the upper 75 m relative to offshore stations. Five nearshore stations with Chl concentrations above the mean (0.12 ± 0.06 μg Chl L^−1^) had corresponding MLDs that were shallower than the DCM, indicating that the enhanced Chl values at the surface were not a result of physical entrainment of DCM Chl at the time of sampling.

In this study, we utilized spatial distributions of various ASVs to gain a deeper understanding of the extent of influence of the observed IME on bacterioplankton communities. First, we categorized ASVs by location they were most abundant. ASVs found in significantly higher abundance (*T*‐test *p* < 0.05, Table 1) at reef stations relative to oceanic upper euphotic stations, averaged greater than a 1% abundance on the reef, and displayed a greater than twofold difference in mean abundance between reef and oceanic stations were defined as ‘reef ASVs’. Likewise, ASVs found in significantly higher abundance (*T*‐test *p* < 0.05) with a twofold increase in abundance in the oceanic upper euphotic stations relative to reef or mesopelagic stations and a >1% mean abundance in oceanic upper euphotic stations were defined as ‘oceanic upper euphotic ASVs’.

**Table 1:** Reef ASVs with reef/oceanic averages and *T*‐test *p*‐values for reef ASVs found in oceanic stations.

Amplicon sequence variant (ASV)	Reef mean abundance (%)	Oceanic (≤75 m) mean abundance (%)	*T*‐test *p*‐value	*T*‐test adjusted *p*‐value
Cyanobacteria_Oxyphotobacteria_Synechococcales_Cyanobiaceae_*Synechococcus*_CC9902_7	6.48 ± 6.17	0.51 ± 1.53	2.95 × 10^−11^	3.06 × 10^−10^
Proteobacteria_Gammaproteobacteria_AlteromoNAdales_Alterornonadaceae_Alteromonas_9	5.48 ± 6.07	0.04 ± 0.14	3.21 × 10^−10^	2.80 × 10^−9^
Proteobacteria_Alphaproteobacteria_Rhodobacterales_Rhodobacteraceae_HIMB11_10	5.31 ± 3.50	0.03 ± 0.17	4.26 × 10^−19^	9.28 × 10^−18^
Proteobacteria_Gammaproteobacteria_Cellvibrionales_Halieaceae_OM60(NOR5)_18	2.90 ± 1.92	0.03 ± 0.15	6.32 × 10^−19^	1.25 × 10^−17^
Bacteroidetes_Bacteroidia_Flavobacteriales_Cryomorphaceae_19	3.41 ± 2.42	0.03 ± 0.15	1.54 × 10^−17^	2.80 × 10^−16^
Bacteroidetes_Bacteroidia_Flavobacteriales_Flavobacteriaceae_NS5 marine group_29	1.87 ± 1.44	0.01 ± 0.10	4.83 × 10^−16^	7.07 × 10^−15^
Bacteroidetes_Bacteroidia_Flavobacteriales_NS9 marine group_47	1.93 ± 1.49	0.02 ± 0.11	4.86 × 10^−16^	7.07 × 10^−15^
Proteobacteria_Alphaproteobacteria_unclassified_54	1.39 ± 2.40	0.03 ± 0.18	1.37 × 10^−5^	6.78 × 10^−5^

**Fig. 4:**
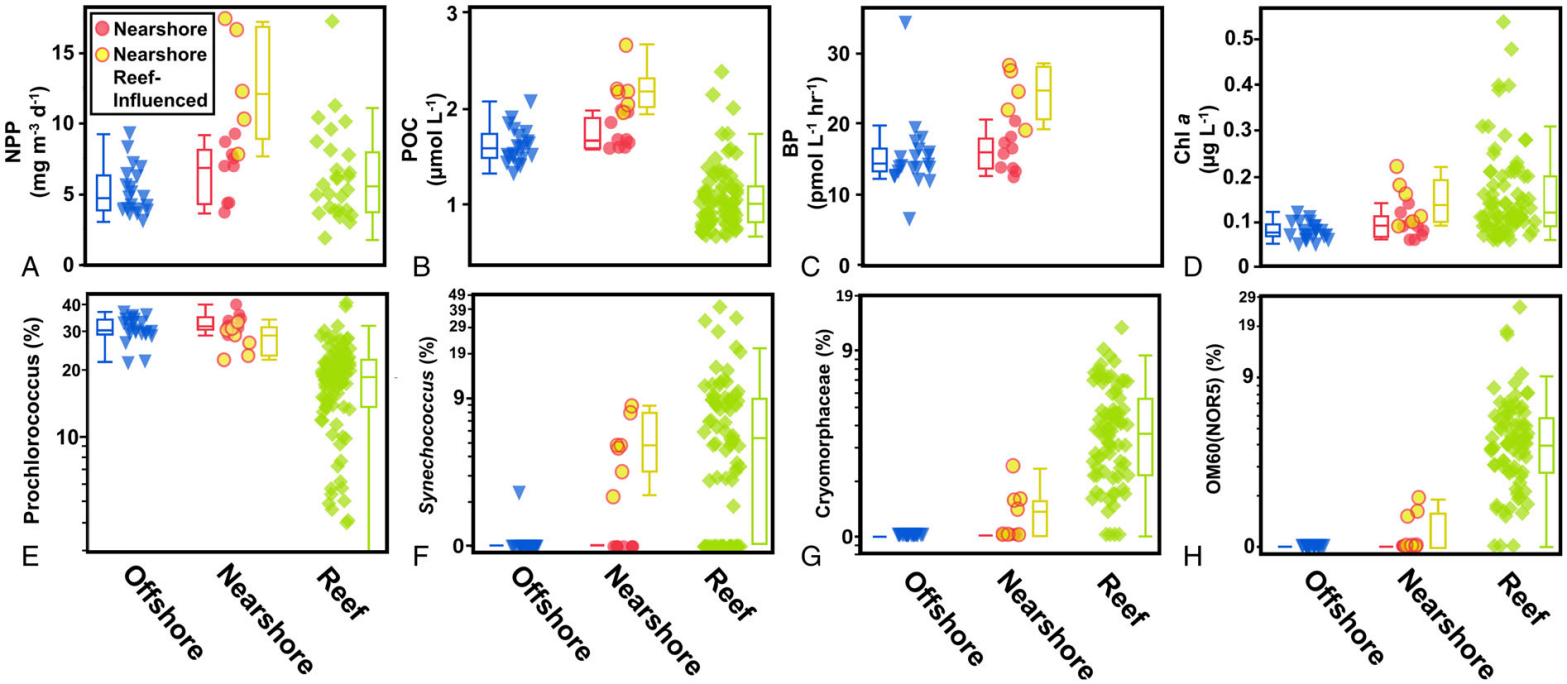
Distribution of key biogeochemical parameters and bacterioplankton taxa among offshore, nearshore and reef surface water habitats. Each symbol is a sample from ≤10 m coloured according to Fig. 1. The top row comprises biogeochemical parameters: Net primary production (NNP; A) particulate organic carbon (POC; B) heterotrophic bacterial productivity (BP; C) and chlorophyll *a* (Chl; D). The bottom row comprises selected abundant bacterioplankton taxa: *Prochlorococcus* (E) *Synechococcus* (F) Cryomorphaceae (G) and OM60(NOR5) (H).

For our analyses, we utilized the nearshore/offshore station groupings previously defined by James *et al*. (2020) as a basic delineation of oceanic stations, then further delineated nearshore stations by the bacterioplankton present. At seven nearshore oceanic stations we observed reef ASVs in the upper euphotic. We defined nearshore oceanic stations with at least one reef ASV present (i.e. > 0%) as ‘nearshore reef‐influenced’ (NRI) stations. No other nearshore stations contained reef ASVs above the level of detection. The reef ASVs that were observed at these NRI stations included *Synechococcus* (#7), Flavobacteriaceae (Cryomorphaceae #19, NS5 #29, and NS9 #47), Alteromonas (#9), OM60(NOR5) (#18), HMIB11 (#10), and unassigned Alphaproteobacteria (#54) (Fig. 4; Table 1). We note that while 21 of our 22 offshore stations did not contain any detectable reef ASVs, there was a single offshore station where *Synechococcus* (#7) was present (Supplementary Fig. 3), indicating that the reef influence could extend beyond the previously defined nearshore stations. The relative abundance of these reef ASVs increased within the reef environment as Chl concentrations and reef residence time increased moving from the forereef to the backreef to the bay (Hench *et al*., 2008) (Supplementary Fig. 4).

NRI stations were found to have significantly higher (one‐way ANOVA, *p* < 0.05) Chl, NPP, POC and BP than both other nearshore stations and offshore stations in the upper 75 m, with the largest differences in mean concentrations found in the upper 10 m (Table 2). Biogeochemical parameters and select ASV abundances in the upper 10 m are visualized in Fig. 4. Additionally, the NRI stations also had a lower mean relative abundance of the most abundant oceanic upper euphotic ASV, *Prochlorococcus* (#1) (27.1 ± 1.6%) than other oceanic stations (31.7 ± 0.7%). Ordination of the microbial community structure in the surface 10 m revealed three distinct clusters; the reef community, a mixture of nearshore and offshore, and the NRI (PERMANOVA *R* ^2^ = 0.27, *p* < 0.001, Fig. 2C). Separation of these three clusters was observed throughout the upper 75 m (Supplementary Fig. 2); however, the degree of difference between NRI stations and other oceanic stations was greatest in the upper 10 m (Fig. 2C) relative to the entire upper 75 m (pairwise PERMANOVA, *R* ^2^ = 0.06 at 10 m and 0.02 in for the upper 75 m, *p* < 0.001). This suggests the cause of the unique communities was due to influence from the surface rather than upwelling from depth, consistent with the hypothesis of James *et al*. (2020) that the DCM was not the source of enhanced Chl at the surface. In the upper 10 m, NRI stations displayed a similar magnitude of difference between other oceanic stations (pairwise PERMANOVA *R* ^2^ = 0.06, *p* = 0.002) and reef stations (pairwise PERMANOVA *R* ^2^ = 0.05, *p* < 0.001). NRI stations thus represent a mixture of oceanic and reef communities, demonstrating connectivity between the reef and nearshore microbial community and biogeochemical response.

**Table 2:** Mean concentrations and ANOVA *p*‐values for chlorophyll (Chl), particulate organic carbon (POC), net primary production (NPP) and heterotrophic bacterioplankton production (BP) at 10 m and in the upper 75 m of offshore, nearshore and nearshore‐modified stations.

	10 m				Upper 75 m			
	Nearshore‐enhanced mean	Nearshore mean	Offshore mean	ANOVA *p*‐value	Nearshore‐enhanced mean	Nearshore mean	Offshore mean	ANOVA *p*‐value
Chl (μg L^−1^)	0.14 ± 0.05	0.09 ± 0.03	0.08 ± 0.01	<0.0001	0.16 ± 0.04	0.10 ± 0.03	0.08 ± 0.02	<0.0001
NPP (μM C d^−1^)	1.07 ± 0.34	0.55 ± 0.17	0.43 ± 0.14	<0.0001	0.71 ± 0.42	0.38 ± 0.21	0.31 ± 0.17	<0.0001
POC (μM C d^−1^)	2.19 ± 0.25	1.73 ± 0.16	1.60 ± 0.18	<0.0001	2.10 ± 0.23	1.73 ± 0.13	1.61 ± 0.17	<0.0001
BP (μM C d^−1^)	0.07 ± 0.01	0.05 ± 0.01	0.05 ± 0.01	0.0012	0.07 ± 0.01	0.05 ± 0.01	0.05 ± 0.01	0.0005

### IME resolved in the deep chlorophyll maximum

**Fig. 5:**
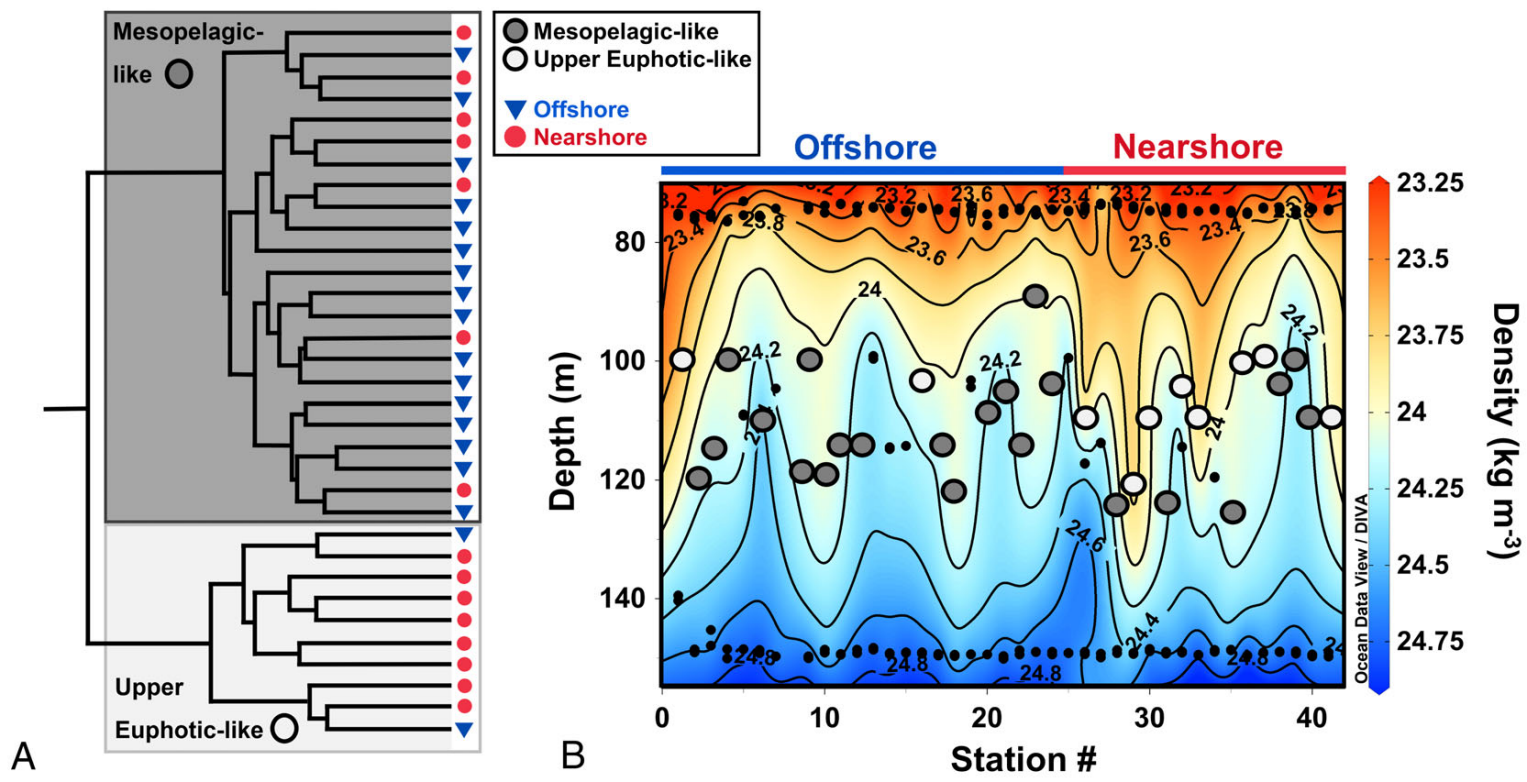
Deep chlorophyll maxima (DCM) samples cluster into two distinct groups according to proximity to nearshore habitats. (A) DCM samples clustered using Ward's method with Bray–Curtis dissimilarity distances; two distinct clusters are resolved and annotated according to their similarity to adjacent shallower (‘upper‐euphotic‐like’; light grey) or deeper (‘mesopelagic‐like’; dark grey) depth communities. (B) Contour plot overlaying sample community types on density isobaths. Small black dots between 90 and 125 m show samples where density measurements were taken but prokaryote community data are absent. Light grey ‘upper euphotic‐like’ communities were found almost exclusively at nearshore (red) stations.

The DCM ranged between 90 and 125 m during the sampling period. There was a larger phylogenetic variability within the DCM and 150 m compared to other depth ranges and reef samples (Supplementary Fig. 5). There was a significant correlation between ordination of the DCM communities (NMDS ordination of Bray–Curtis distances) and water mass captured within the density surfaces between 23.6 and 24.5 kg m^−3^ (PERMANOVA *R* ^2^ = 0.150, *p* < 0.001). This suggests the shoaling and deepening of these isopycnals through the DCM influenced the composition of the DCM microbial community structure. A clear bifurcation of DCM samples was observed (Fig. 5A) when DCM samples were hierarchically clustered with Ward's minimum variance method using Bray–Curtis dissimilarity values. ASVs found only in one DCM sample were removed from multivariate analyses to focus on relative abundance changes of cosmopolitan DCM bacterioplankton due to the high number of individual DCM station ASVs. The two clear groupings ordinated separately (PERMANOVA *R* ^2^ = 0.15, *p* < 0.001, Supplementary Fig. 6) and can be broadly characterized as influenced by ‘upper‐euphotic‐like’ or more ‘mesopelagic‐like’ communities (Fig. 5A). Upper‐euphotic‐like communities had a smaller magnitude of difference with upper euphotic communities (pairwise PERMANOVA *R* ^2^ = 0.19, adjusted *p* = 0.006) than mesopelagic‐like communities had with upper euphotic communities (pairwise PERMANOVA *R* ^2^ = 0.45, adjusted *p* = 0.006). Likewise, mesopelagic‐like communities had a smaller magnitude of difference with 150 m communities (pairwise PERMANOVA *R* ^2^ = 0.21, adjusted *p* = 0.006) than upper‐euphotic‐like communities had with 150 m communities (pairwise PERMANOVA *R* ^2^ = 0.24, adjusted *p* = 0.006) (Supplementary Fig. 6). Samples within the mesopelagic‐like group occupied significantly denser water masses (mean = 24.14 ± 0.11 kg m^−3^) than samples within the euphotic‐like group (mean = 23.90 ± 0.17 kg m^−3^, *T*‐test *p* = 0.0014) (Fig. 5B). Thus, euphotic‐like communities coincided with the vertical displacement of less‐dense isopycnals to deeper depths and mesopelagic‐like communities coincided with uplift of denser isopycnals to shallower depths (Fig. 5B). Additionally, mean oxygen and DOC concentrations were significantly lower for waters surrounding the mesopelagic‐like group (O_2_ = 196.7 ± 2.7 μM O_2_; DOC = 62.1 ± 4.4 μM C) than for those surrounding the upper euphotic‐like group (O_2_ = 199.7 ± 2.1 μM O_2_; DOC = 66.0 ± 3.5 μM C, *T*‐test *p* = 0.0027 and *p* = 0.0173). Pairwise PERMANOVA results and mean values for density, oxygen and DOC are listed in Supplementary Table 3. No significant difference in nutrient concentrations (nitrate + nitrite, phosphate) was observed between nearshore and offshore stations or between upper euphotic‐like and mesopelagic‐like communities, suggesting that nutrient dynamics were not primary drivers in community differentiation in the DCM.

The physical and chemical differences between the two DCM groupings suggest they are influenced by distinct water masses. Mesopelagic‐like communities had an increased relative abundance of SAR86, *Prochlorococcus* (#59), Flavobacteriaceae, SAR 202 (#154) and SAR 406 (#31). Euphotic‐like samples displayed enhanced abundance of ASVs found in higher abundance in the upper 75 m, including *Prochlorococcus* (#1), AEGEAN‐169 (#8, 52), SAR202 (ASV #60, 99) and SAR11 (#3, 4, 6, 14) (Supplementary Table 2). These are not reef taxa found present in NRI stations – they are taxa found in high relative abundance in the upper 75 m of all oceanic stations. While mesopelagic‐like DCM communities were also found at nearshore stations, the euphotic‐like DCM communities (light grey circles) were found almost exclusively at nearshore stations (red stations) that lie within 15 km of either Mo′orea or Tahiti (Fig. 5A and B). This indicates that proximity to the islands influenced the downward displacement of lower density isopycnals that resulted in the DCM of those stations being comprised of more surface‐like taxa. Thus, IME appears to influence microbial community structure as deep as the DCM.

### Bacterioplankton variability in the mesopelagic

PERMANOVAs were used to determine if nearshore and offshore samples ordinated separately both within the entire upper mesopelagic and within each discrete depth throughout the upper mesopelagic (nominally 150, 200, 250, 300 and 500 m). While mesopelagic communities clearly separated between discrete depths (Fig. 2A and B), these analyses did not reveal any significant difference in ordination with regard to proximity to shore for any of the mesopelagic depths. Thus, we were unable to detect an IME on microbial community structure deeper than the DCM.

**Fig. 6:**
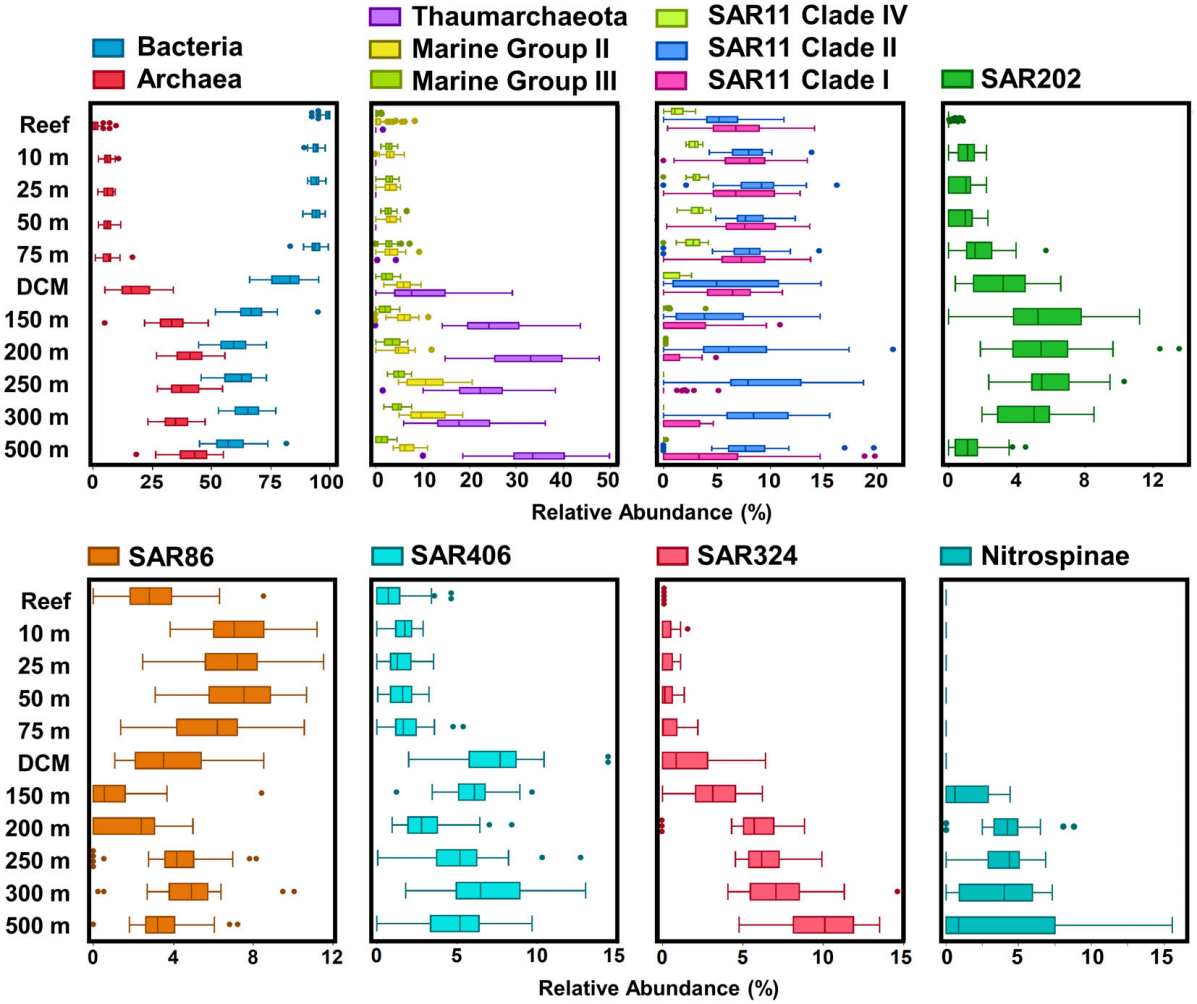
Relative abundances of major mesopelagic bacterioplankton taxa at each discrete depth horizon sampled.

Hierarchical clustering demonstrated clear vertical stratification of bacterioplankton community structure within the mesopelagic (150–500 m) (Fig. 2). At the domain level, the proportion of Archaea in the overall bacterioplankton community increased with depth and reached a maximum in relative abundance at 200 and 500 m in oceanic stations (Fig. 6) comprising up to 55% of total community structure. Marine Group II (MGII) Euryarchaeota averaged between 3% and 6.5% of the overall community at all depths from the surface to 200 m, while Marine Group III (MGIII) averaged approximately 3% through the euphotic zone (Fig. 6). Deeper within the mesopelagic zone, the relative abundance of MGII increased to a maximum of 10.9% of the overall community structures by 250–300 m. MGIII reached its highest average relative abundance of 5.1% at 250 m. Thaumarchaeota were resolved in only two samples shallower than the DCM (two samples at 75 m). However, the relative contribution of Thaumarchaeota to the total bacterioplankton community increased with depth starting within the DCM (90–125 m) and reaching maximal relative contributions of 34% and 32% at 200 and 500 m respectively (Fig. 6). The two deep Thaumarchaeota maxima were comprised of distinct ASVs (ASVs #18, 51 and 161 at 300 m, ASVs #15, 32, 48 and 195 at 500 m, Fig. 3).

Within the bacterial domain, the most abundant taxa in the mesopelagic included members of the SAR11, SAR202, SAR86, SAR406 and SAR324 clades (Figs 3 and 6). Within the SAR11 Clade, subclades I and IV decreased in relative abundance from surface to mesopelagic, while members of SAR11 Ib increased in relative abundance from upper euphotic to DCM and mesopelagic. This is consistent with previously understood SAR11 distributions (Giovannoni, 2017). Average relative abundance of SAR11 subclade II was not significantly different between upper euphotic and mesopelagic depths; however, within the clade individual ASVs showed variable depth distributions. SAR202 was present at low relative abundances in the top 50 m, averaging less than 2% at all depths. Beginning at 75 m, the relative abundance of SAR202 began to increase with depth, reaching greatest relative abundance between 150 and 250 m. SAR86 was found in highest relative abundance in the upper 50 m of oceanic stations before declining to its lowest relative abundance at 150 m, then increasing again between 200 and 500 m. The SAR86 ASVs that occupied the upper euphotic and the mesopelagic were distinct (ASVs #20, 34, 36, 39 in upper euphotic, ASVs # 27, 79 in mesopelagic, Fig. 3; Supplementary Table 1). Similar to SAR202, SAR406 maintained a low relative abundance in the upper euphotic, with two peaks in relative abundance in the DCM and 300 m. SAR324 was in low abundance in the upper euphotic, but increased with depth, peaking at 500 m (Fig. 6).

## Discussion

Altered physical dynamics, biogeochemical patterns and enhanced biological activity observed for stations within the nearshore oceanic environment provide evidence that is consistent with the phenomenon known as an IME (Andrade *et al*., 2014; Doty & Oguri, 1956). James *et al*., 2020 analysed an extensive biogeochemical data set collected from a 51 km × 67 km grid encompassing the Society Islands of Mo'orea and Tahiti in the South Pacific Subtropical Gyre. They proposed that an IME influenced the biogeochemical constituents around the islands of Mo′orea and Tahiti, with nearshore stations showing enhanced levels of NPP, Chl, BP and POC in the upper euphotic zone of the water column. They hypothesized that advection of reef influenced water may have contributed to those nearshore features. However, in the absence of a chemical tracer or biological marker they were unable to establish a direct connection between the reef water and nearshore ‘hot spots’. In addition, the high variability associated with bulk measurements of biological rates (NPP and BP) and concentrations of biogeochemical variables limited their ability to resolve the vertical extent of the potential IME beyond the surface 75 m.

In the present study we used 16S rRNA gene metabarcoding data, collected concurrently with the previously reported biogeochemical measurements (James *et al*., 2020) to assess whether the metabarcoding data provided a useful biological tracer of an IME. We explored the use of bacterioplankton ASVs as biological tracers to better elucidate the sources of the observed enhancement of key biogeochemical constituents at nearshore stations. More specifically, we sought to determine whether the metabarcoding data could help resolve the potential influence of an IME on the distribution, composition and connectivity of microbial communities along horizontal (reef to offshore) gradients as well as over depth, extending from the upper euphotic zone through the DCM and into the upper mesopelagic zone.

### Island Mass Effect at the surface

Horizontal advection of reef water with elevated productivity and nutrient concentrations was hypothesized as a primary mechanism supporting the IME (James *et al*., 2020). An alternative hypothesis is that the elevated surface Chl and NPP at the NRI stations resulted from entrainment of photoautotrophs and nutrients from the DCM and upper mesopelagic, bringing a greater Chl signature to the surface or stimulating primary production. While previous studies have observed upwelling‐induced IMEs near deep‐sea islands (Furuya *et al*., 1986; Hasegawa *et al*., 2004; Heywood *et al*., 1990), two lines of evidence rule out upwelling as a source for enhanced NPP and surface Chl concentrations around Mo′orea and Tahiti. First, the study area was a relatively well‐stratified system, characteristic of Pacific subtropical gyres (Karl & Church, 2014, 2017; Wilson *et al*., 2015). Five nearshore stations with enhanced Chl were found to have MLD shallower than 75 m (James *et al*., 2020), precluding entrainment of Chl from the DCM or nutrients from upper mesopelagic into the upper euphotic zone. Second, bacterioplankton ASVs in the surface 75 m were distinct from those in the DCM (Fig. 3). For example, the ASVs found in highest relative abundance in the DCM (e.g. *Prochlorococcus* #44, #59, #131, SAR406 #31, SAR11 Ib #69, SAR324 #113) were absent from samples collected above 75 m. We observed distinct depth ranges for ASVs through the water column, with one notable example being the separation of three *Prochlorococcus* ASVs through the euphotic zone, all members of strain MIT9313. All *Prochlorococcus* ASVs found in this dataset have previously been reported in the subtropical Pacific (Berube *et al*., 2018). The stratification of *Prochlorococcus* ecotypes in this dataset reflects subclade adaptations to varying light and nutrient fields within the euphotic zone (Biller *et al*., 2014; Braakman *et al*., 2017; Durand *et al*., 2001; Rocap *et al*., 2003), providing unique signatures for tracking the vertical displacement of water masses.

Consistent with the hypothesis of horizontal advection of modified reef water, we observed that bacterioplankton ASVs found in significantly and substantially higher relative abundance in reef stations were located at oceanic stations with enhanced Chl, NPP, BP and POC (Table 1; Fig. 4), suggesting advection of water from the reef to the nearshore oceanic was a potential source for altered biogeochemistry and biology at these NRI stations. All individual reef taxa except *Synechococcus* (#7) average <1% of overall abundance in oceanic stations, suggesting they are not the major drivers of community structure ordination. However, these specific ASVs can serve as biological tracers connecting the biology of the reef environment with that of surrounding oceanic stations. The presence of reef‐associated ASVs and decreased relative abundance of the oligotrophic cyanobacterium *Prochlorococcus #1* at NRI stations are associated with a separation in ordination from other nearshore stations (Fig. 2C). The similar magnitude of difference in ordination NRI stations displayed with other oceanic and reef stations highlights the gradient in community between reef and offshore oceanic environments that mapped onto the gradients of Chl and productivity established by James *et al*. (2020).

The horizontal advection of reef and inshore waters has been observed around atolls in the Northwestern Hawaiian Islands (Gove *et al*., 2016) and around Rangiroa Atoll in the French Polynesian Tuamotu archipelago (Vollbrecht *et al*., 2021), both of which have pronounced IMEs. Ocean swells can pump substantial amounts of water over the fore and backreefs of these islands and atolls that then flow through the reef system and exit through reef passes (Aucan *et al*., 2012), similar to the flow around Mo′orea (Hench *et al*., 2008). Wave forcing across atolls acts as a highly efficient flushing mechanism, advecting detritus and other nutrient sources from the coral reef into the surrounding nearshore oceanic environment (Callaghan *et al*., 2006). Rapid flushing rates associated with coral reef systems can exceed rates of nutrient assimilation by the coral reef benthic community (Atkinson, 2011), providing a mechanism by which the nearshore oceanic environment can experience higher nutrient input and higher phytoplankton biomass than the offshore environment. The coral reefs around Mo′orea experience a rapid flushing rate with a backreef residence time of hours to days (Hench *et al*., 2008; Knee *et al*., 2016); thus, advection of water from the coral reef to the nearshore oceanic environment is a plausible source of increase in biological productivity near the island. Additionally, the observed weak counterclockwise flow around the island of Mo′orea could act as a mechanism for retention of advected reef water in the surrounding nearshore oceanic environment (James *et al*., 2020; Leichter *et al*., 2013). However, with the data collected, it remains unknown whether the presence of reef ASVs in the nearshore oceanic environment is solely due to advection and subsequent dilution of reef ASVs, or whether advection of chemical subsidies from the reef allowed for growth of reef‐like prokaryotic populations at NRI stations. Thus, further discussion on metabolic potential and activity of these reef taxa in the oceanic environment without additional supporting data would be limited to conjecture.

### Evidence of IME over depth

The metabarcoding data from the present study revealed several distinct bacterioplankton taxa typically observed within the upper euphotic zone, such as *Prochlorococcus* #1, AEGEAN‐169 (#8), SAR202 (ASV #60, 99) and SAR11 (#3, 4, 6, 13, 14), occupying comparatively low density, warm water in the DCM at stations near the islands. This suggests island‐influenced vertical displacement of upper euphotic water occurred around time of sampling. The cause of this downwelling cannot be determined from data in the present study; however, surface currents and eddies have been found to vertically displace local surface‐derived organic matter, oxygen and biological activity to mesopelagic depths in oceanic systems (Benitez‐Nelson & McGillicuddy, 2008; McGillicuddy *et al*., 1999, 2007; Nelson *et al*., 2014). At time of sampling, a predominance of mesoscale anticyclonic flow was observed in the region around Mo′orea and Tahiti (James *et al*., 2020). On a smaller scale, a weak counterclockwise flow has been observed around Mo′orea, and was present at time of sampling (James *et al*., 2020; Leichter *et al*., 2013). This anticyclonic flow likely resulted in the observed elevated sea surface height around Mo′orea and Tahiti (James *et al*., 2020), causing downward displacement of the pycnocline, depressing the nutricline and upper euphotic biogeochemistry (McGillicuddy *et al*., 1999). We suggest that upper euphotic biology was displaced downward because of this anticyclonic flow, with the islands acting as a physical barrier for the buildup and vertical depression of nearshore upper euphotic water masses. Nutrient concentrations did not vary significantly between upper euphotic‐like and mesopelagic‐like DCM communities, suggesting that changes in nutrient regimes were not a primary driver for differentiation of bacterioplankton communities within the DCM. Therefore, influence of an IME on the DCM was observed through downward displacement of less dense isopycnals with enhanced oxygen concentration and increased relative abundance of upper euphotic taxa.

In the mesopelagic, clear clustering of bacterioplankton taxa was observed, with ASVs displaying variation in relative abundance by depth (Figs 2A, B and 3). The vertical stratification of bacterioplankton taxa from the upper euphotic into the mesopelagic observed in the present study (Fig. 3) is consistent with previous findings in the stratified ocean systems (Bryant *et al*., 2016; DeLong *et al*., 2006; Field *et al*., 1997; Giovannoni *et al*., 1990; Konstantinidis *et al*., 2009; Mende *et al*., 2017). While the mesopelagic microbial communities clearly separated between discrete depths, further multivariate analyses were not able to resolve any additional significant relationship with distance to shore. Thus, 16S rRNA gene metabarcoding as a tracer of IME was not useful at depths deeper than the DCM with the resolution of the present dataset.

Wide variability in core properties of microbial genomes such as GC content and genome size has been found among different archaea and bacteria (McCutcheon & Moran, 2011; Ochman & Davalos, 2006). A sharp transition zone in this genetic variability is reported to occur at the base of the euphotic zone correlating with changes in the surrounding energy and nutrient fields, supporting the hypothesis that nutrient limitation is a central driver in the evolution of genomic properties of bacterioplankton (Mende *et al*., 2017). IMEs are often observed because of input of limiting nutrients into the surrounding environment, whether by riverine runoff (Anderson *et al*., 2002), advection by wave‐forcing (Callaghan *et al*., 2006), groundwater discharge (Street *et al*., 2008), near‐island upwelling (Gove *et al*., 2006; Hamner & Hauri, 1981), or turbulence from eddies and wakes on the downstream side of the island (Coutis & Middleton, 1999; Hernández‐León, 1991; Heywood *et al*., 1990). Therefore, we suggest the influence of an IME is most obvious in populations above the nutricline where horizontal and vertical delivery of limiting nutrients can influence microbial activity and resulting community structure. Below the nutricline, presence of limiting nutrients in the upper euphotic such as nitrogen and phosphorus no longer become the primary limitation for microbial growth, and therefore addition of nutrients from processes near the islands may not alter microbial growth or composition.

The dark ocean (below 200 m depth) harbours approximately 65% of all bacterioplankton (Whitman *et al*., 1998), and dark ocean chemoautotrophic DIC fixation has been suggested to be in the same order of magnitude as heterotrophic activity, equalling 15%–53% of phytoplankton export production in the North Atlantic (Reinthaler *et al*., 2010). Chemoautotrophic capabilities have been found in multiple common dark ocean taxa, including taxa abundant below the DCM in this dataset, such as ammonia‐oxidizing Thaumarchaeota (Wuchter *et al*., 2006), nitrite‐oxidizing Nitrospinae (Lücker *et al*., 2013) and sulfur‐oxidizing SAR324 (Swan *et al*., 2011). All these taxa were in low abundance or below detection in the upper 75 m and increased in abundance at or below the DCM. These taxa distribution patterns exemplify the shifting metabolic functions and nutritional strategies from photoautotrophy in the sunlit euphotic to chemoautotrophy in the dark ocean.

## Conclusion

Our study utilizes 16S rRNA gene metabarcoding data to trace origins of observed physical and biogeochemical variability around the islands of Mo′orea and Tahiti. We present clear evidence of an IME, with reef bacterioplankton found in the oceanic environment at nearshore stations with enhanced Chl, NPP, POC and BP. This suggests alteration of oceanic water by the island's reefs and the subsequent advection and retention of this microbially and biogeochemically altered water in the nearshore oceanic environment is likely a major mechanism contributing to the IME observed. Within the DCM, microbial communities with greater abundances of upper euphotic bacterioplankton were found almost exclusively in the nearshore environment. While the mechanisms for this observation are not clear, it is possible that anticyclonic flow around the island could provide a mechanism for downwelling into the DCM. Within the mesopelagic, no IME was observed. We hypothesize that island proximity no longer has a significant impact on the conditions necessary for microbial growth below the nutricline, as nutrients no longer become a limiting factor to growth. Our results demonstrate the utility of metabarcoding data as a biological tracer for physical and biogeochemical variability and provide further insight into the extent and influences of phenomena such as mesoscale perturbations (Nelson *et al*., 2014) and IMEs.

## Supporting information


**Supplementary Fig. 1.** Reef sampling locations on the north (a), west (b), and east (c) sides of Mo′orea. Light grey shading indicates the reef platform, nominally 3 m deep. Dark grey shading indicates land.

**Supplementary Fig. 2.** Non‐metric multidimensional scaling (NMDS) ordination of bacterioplankton communities in the top 75 m in the reef and oceanic environments

**Supplementary Fig. 3.** Contour plots of chlorophyll *a* (a) *Synechococcus* relative abundance (b) OM60(NOR5) relative abundance (c) and Cryomorphaceae (d) relative abundance in the upper 150 m of the water column in the pelagic environment. Black dots indicate sites of sample collection. Colour boxes indicate offshore (blue) and nearshore (red) stations as in Fig. 1.

**Supplementary Fig. 4.** Change in chlorophyll *a* (Chl *a*), *Prochlorococcu*s, *Synechococcus*, Cryomorphaceae, and OM60 (NOR5) in the surface 10 m along NMDS axis 1.

**Supplementary Fig. 5.** The DCM exhibits greater microbial community variation than other depth intervals. Panel (a): Heatmap of pairwise microbial community dispersion. While there were minimal differences in multivariate dispersion of microbial communities among upper euphotic samples (above 90 m) or among mesopelagic samples (below 200 m) (black dashed lines), dispersion at the DCM (90–135 m) and 150 m was significantly greater than all other samples (white dashed lines). Panel (b): The difference in dispersion between DCM/150 m samples and other depth intervals was greater than differences among other depth intervals; for each depth pairwise differences in dispersion with other depths are represented as symbols (black if significant, grey if not significant by Tukey *post hoc p* < 0.05 on dispersion ANOVA) – asterisks represent pairwise differences from the DCM and open circles are the pairwise difference between DCM and 150 m (note that asterisks are generally the greatest pairwise difference in dispersion).

**Supplementary Fig. 6.** Non‐metric multidimensional scaling (NMDS) ordination of bacterioplankton communities at 75 m, 150 m, and in the mesopelagic‐like and upper euphotic‐like groups in the deep chlorophyll maximum (DCM) communities. Upper euphotic‐like DCM communities cluster more closely to communities at 75 m than 150 m, and mesopelagic‐like DCM communities cluster more closely to communities at 150 m than 75 m.

**Supplementary Fig. 7.** Ranges of relative abundances that correspond to the standardized heatmap coloration for each bacterioplankton ASV shown in Fig. 3.

**Supplementary Table 1.** Mean relative abundances and standard deviations for ASVs with an abundance greater than 4% in three samples, or 8% in one sample.

**Supplementary Table 2.** Mean relative abundances for DCM ASVs enhanced in Mesopelagic‐like and Upper Euphotic‐like stations with *T*‐test *p*‐values.

**Supplementary Table 3.** Mean density, oxygen and DOC concentrations alongside pairwise PERMANOVA *R* ^2^ and *p*‐values for the mesopelagic‐like and upper euphotic‐like DCM groups.
