# Supplementary figures and images for: Bacterioplankton communities reveal horizontal and vertical influence of an Island Mass Effect
Source: Environ Microbiol. 2022 Jun 12;24(9):4193–208. doi: 10.1111/1462-2920.16092 (PMC9796716; doi:10.1111/1462-2920.16092)

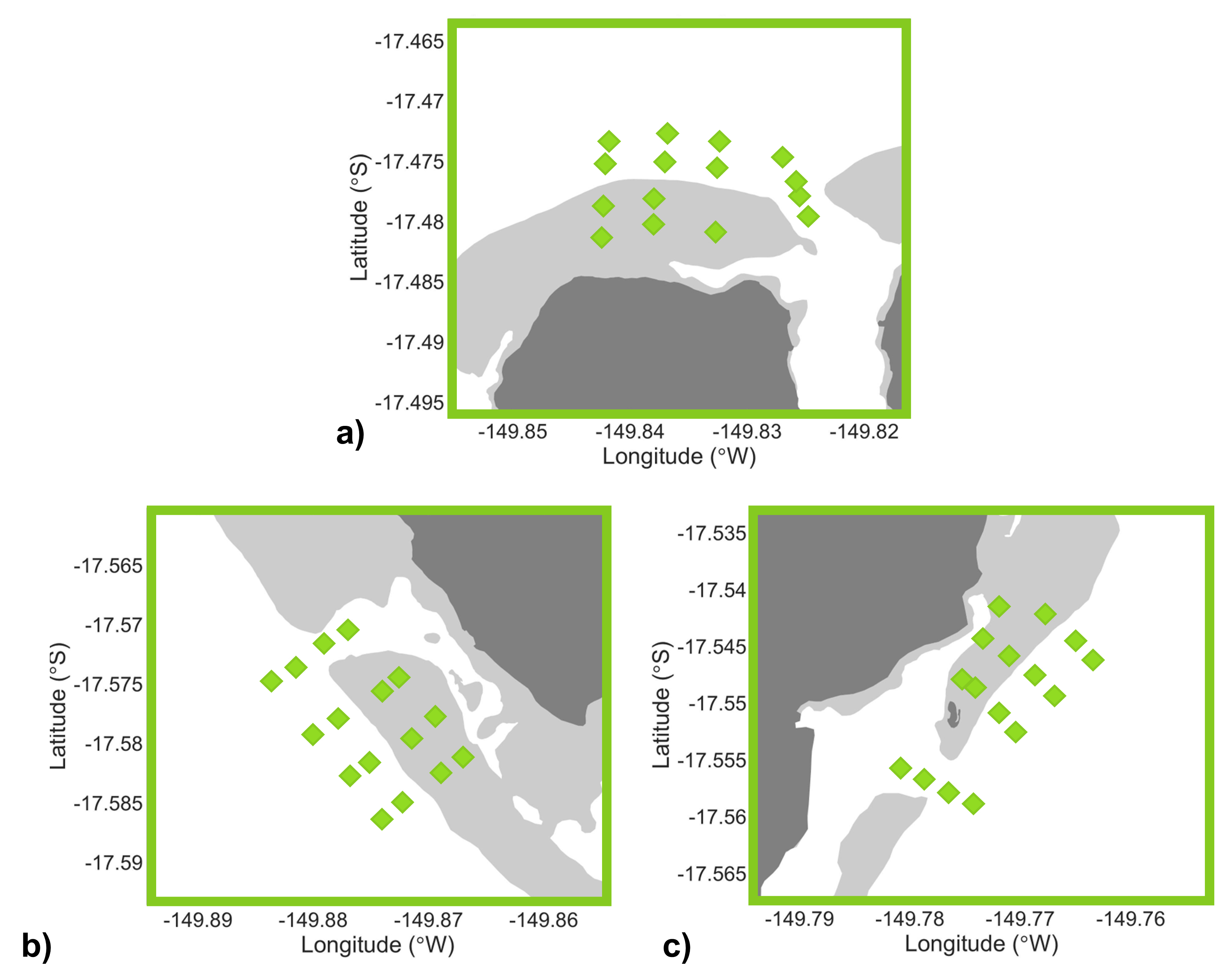

Supplement: Supplementary file 1 — Supplementary Fig. 1. Reef sampling locations on the north (a), west (b), and east (c) sides of Mo′orea. Light grey shading indicates the reef platform, nominally 3 m deep. Dark grey shading indicates land. [file EMI-24-4193-s006.tif]

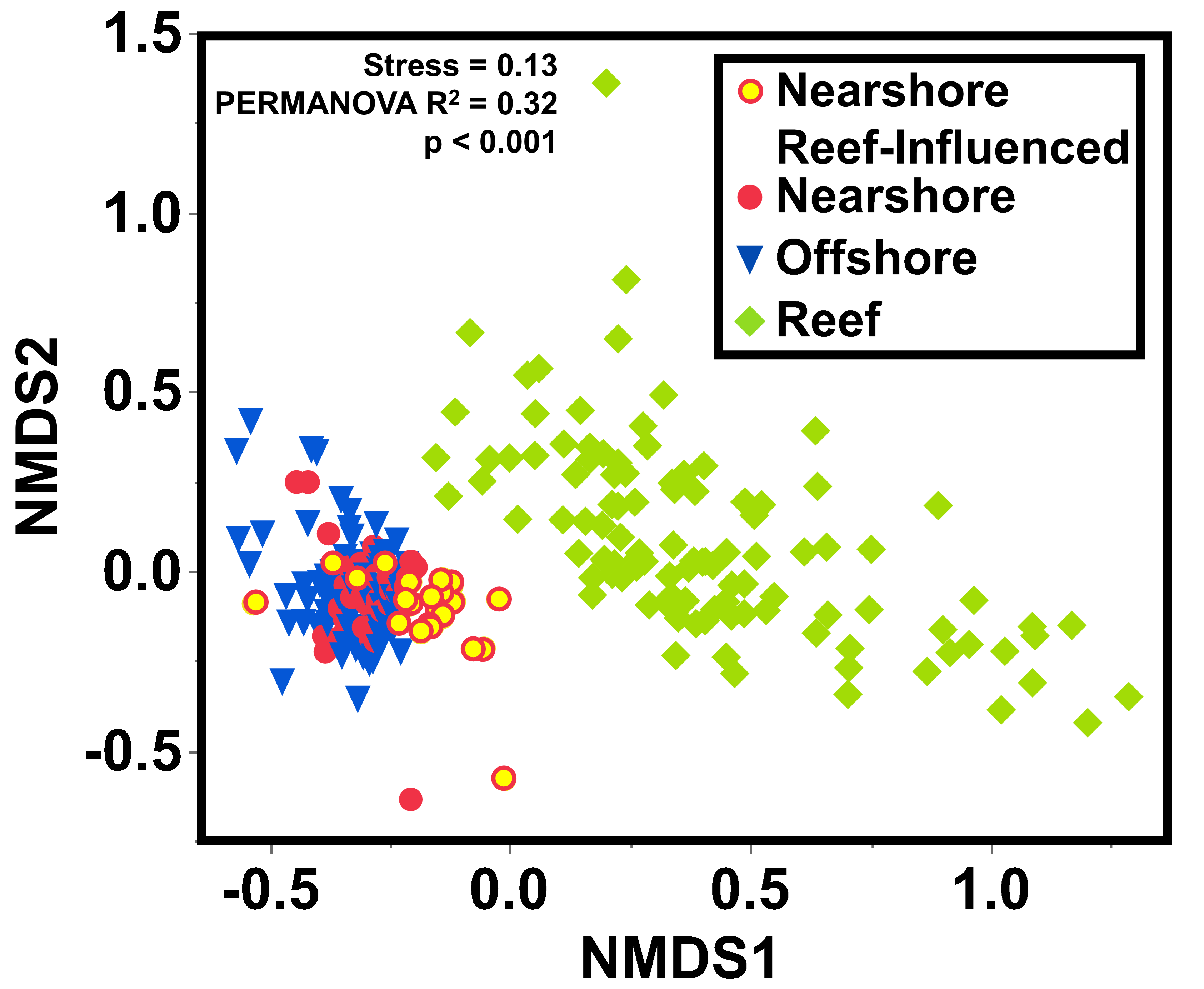

Supplement: Supplementary file 2 — Supplementary Fig. 2. Non‐metric multidimensional scaling (NMDS) ordination of bacterioplankton communities in the top 75 m in the reef and oceanic environments [file EMI-24-4193-s007.tif]

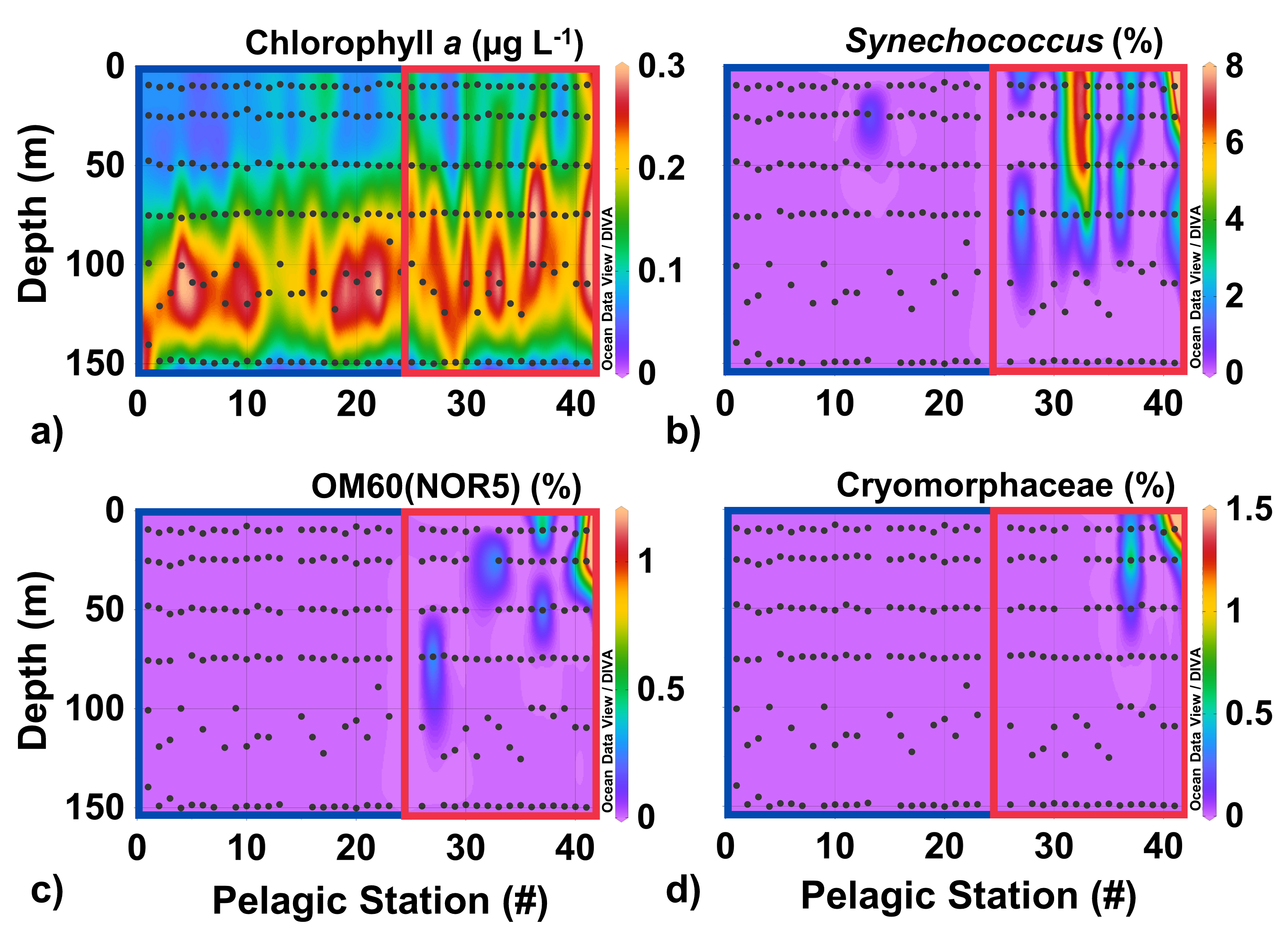

Supplement: Supplementary file 3 — Supplementary Fig. 3. Contour plots of chlorophyll a (a) Synechococcus relative abundance (b) OM60(NOR5) relative abundance (c) and Cryomorphaceae (d) relative abundance in the upper 150 m of the water column in the pelagic environment. Black dots indicate sites of sample collection. Colour boxes indicate offshore (blue) and nearshore (red) stations as in Fig. 1. [file EMI-24-4193-s003.tif]

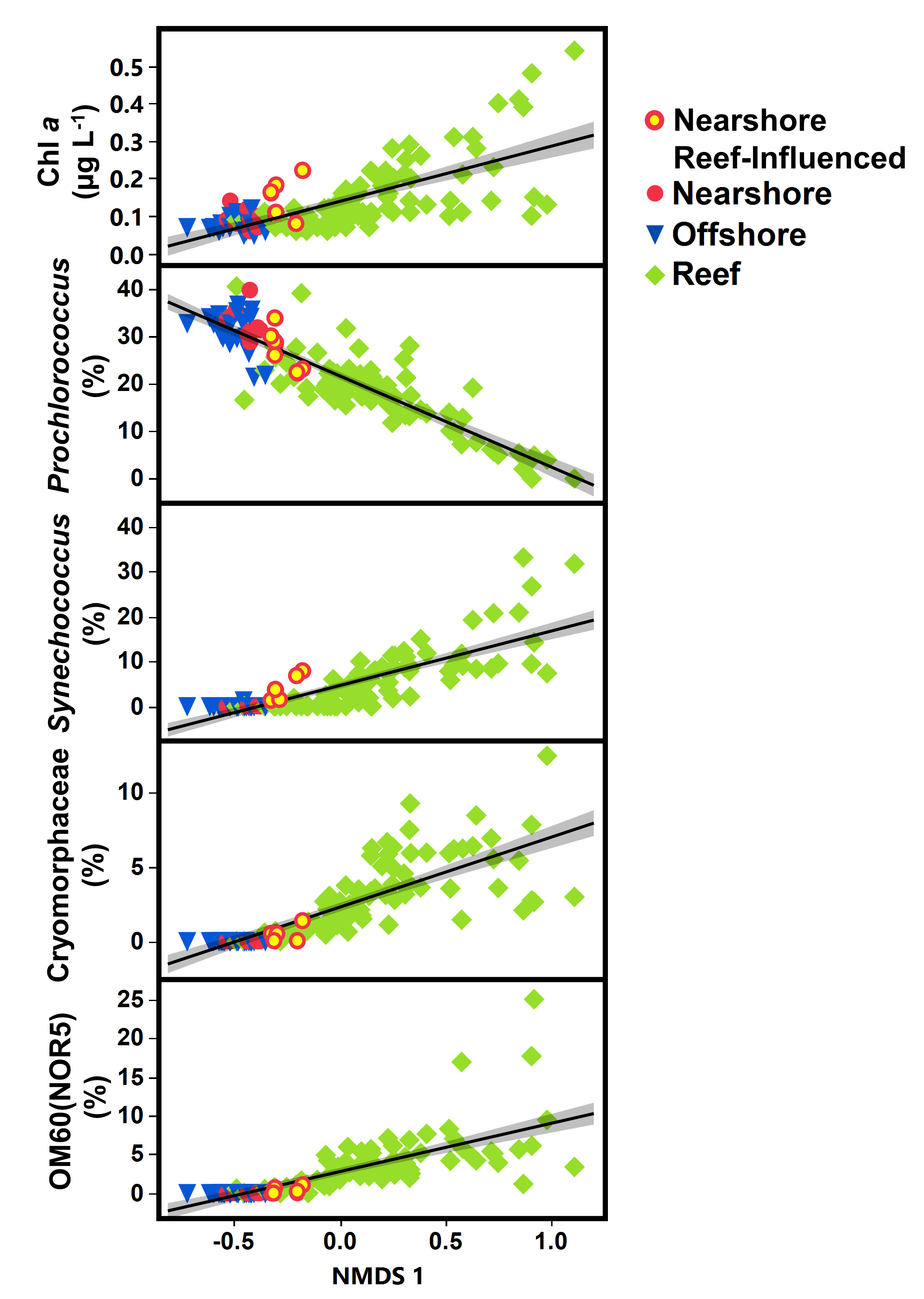

Supplement: Supplementary file 4 — Supplementary Fig. 4. Change in chlorophyll a (Chl a), Prochlorococcus, Synechococcus, Cryomorphaceae, and OM60 (NOR5) in the surface 10 m along NMDS axis 1. [file EMI-24-4193-s008.tif]

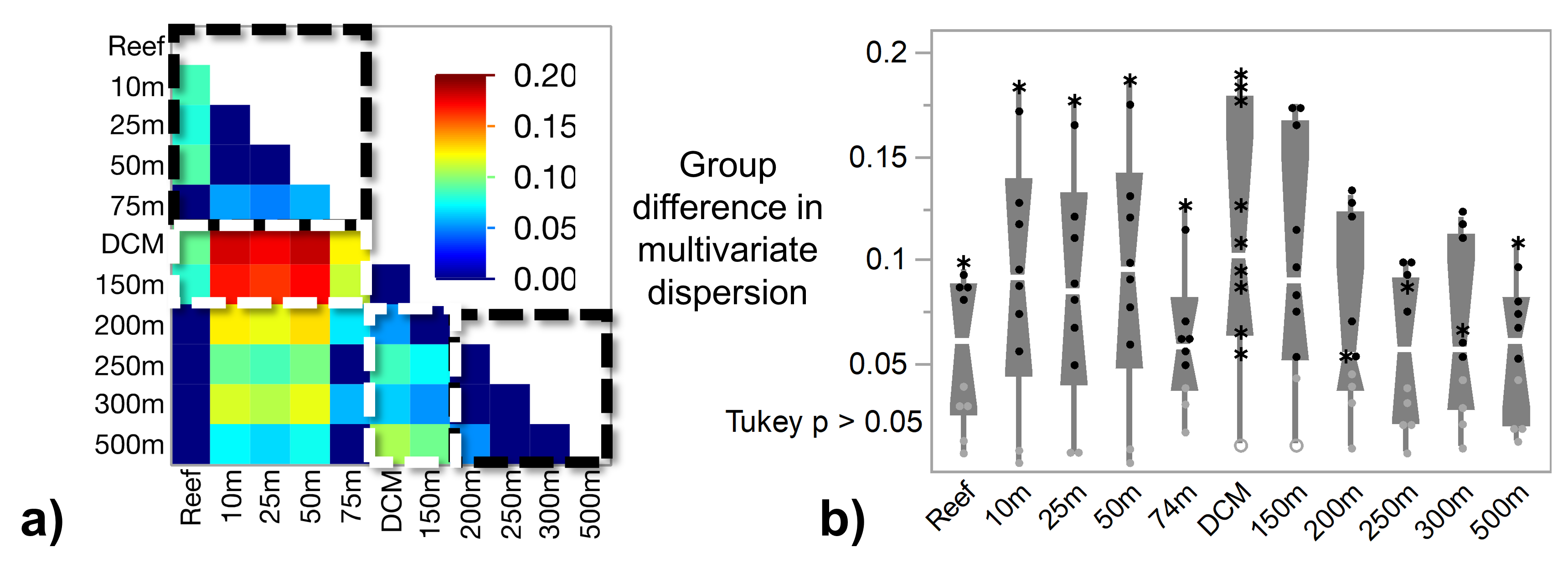

Supplement: Supplementary file 5 — Supplementary Fig. 5. The DCM exhibits greater microbial community variation than other depth intervals. Panel (a): Heatmap of pairwise microbial community dispersion. While there were minimal differences in multivariate dispersion of microbial communities among upper euphotic samples (above 90 m) or among mesopelagic samples (below 200 m) (black dashed lines), dispersion at the DCM (90–135 m) and 150 m was significantly greater than all other samples (white dashed lines). Panel (b): The difference in dispersion between DCM/150 m samples and other depth intervals was greater than differences among other depth intervals; for each depth pairwise differences in dispersion with other depths are represented as symbols (black if significant, grey if not significant by Tukey post hoc p < 0.05 on dispersion ANOVA) – asterisks represent pairwise differences from the DCM and open circles are the pairwise difference between DCM and 150 m (note that asterisks are generally the greatest pairwise difference in dispersion). [file EMI-24-4193-s005.tif]

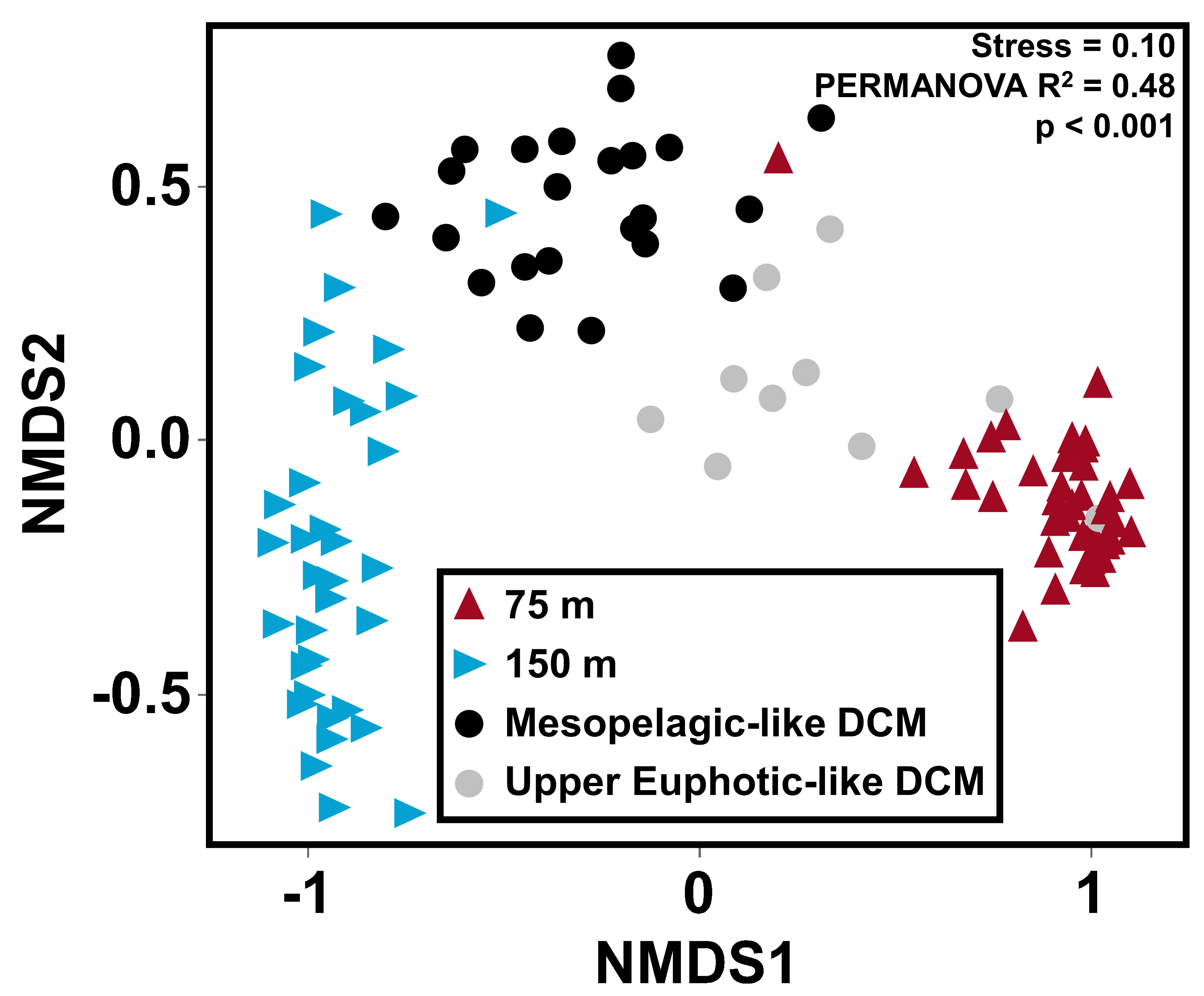

Supplement: Supplementary file 6 — Supplementary Fig. 6. Non‐metric multidimensional scaling (NMDS) ordination of bacterioplankton communities at 75 m, 150 m, and in the mesopelagic‐like and upper euphotic‐like groups in the deep chlorophyll maximum (DCM) communities. Upper euphotic‐like DCM communities cluster more closely to communities at 75 m than 150 m, and mesopelagic‐like DCM communities cluster more closely to communities at 150 m than 75 m. [file EMI-24-4193-s009.tif]

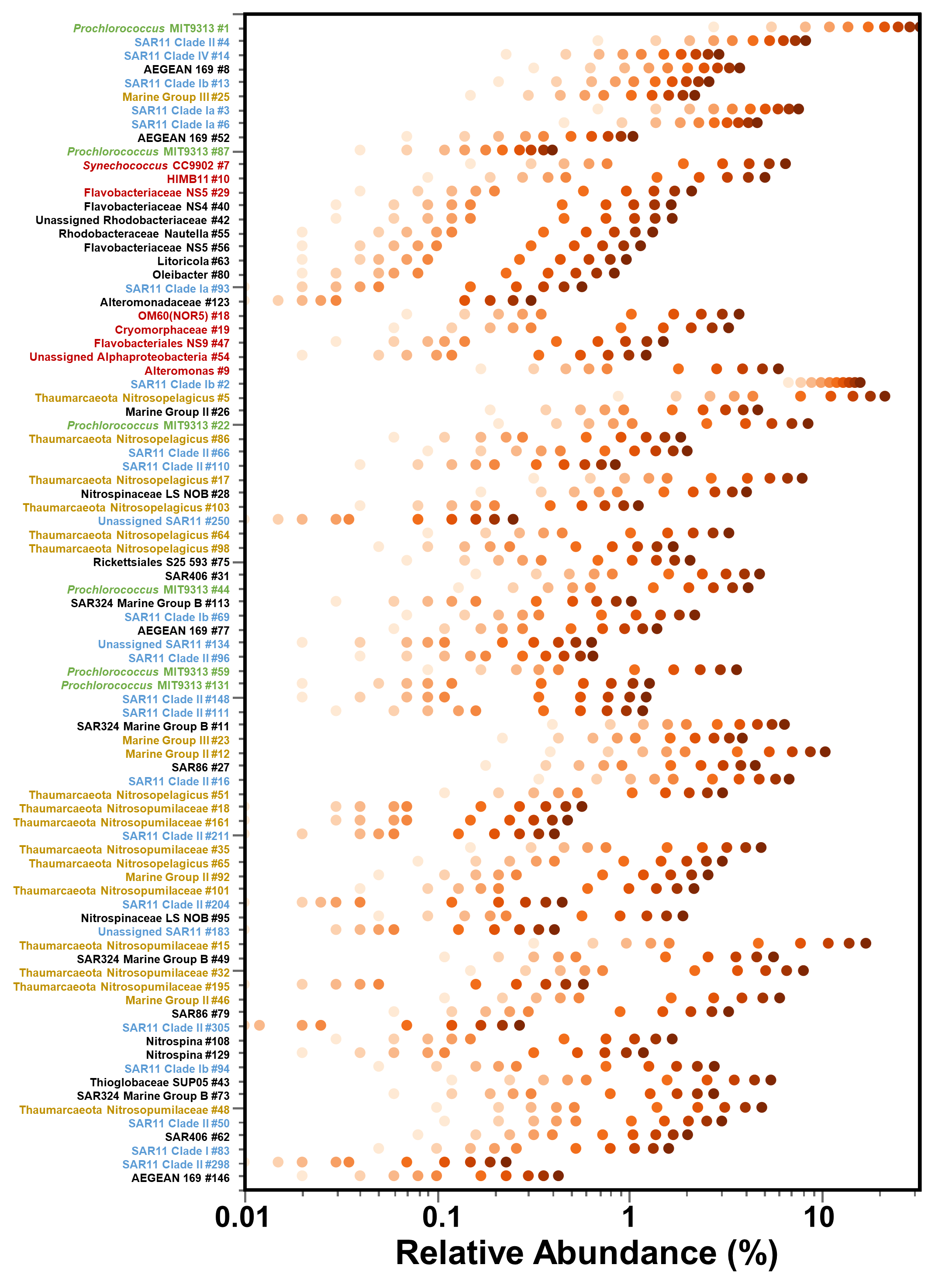

Supplement: Supplementary file 7 — Supplementary Fig. 7. Ranges of relative abundances that correspond to the standardized heatmap coloration for each bacterioplankton ASV shown in Fig. 3. [file EMI-24-4193-s001.tif]

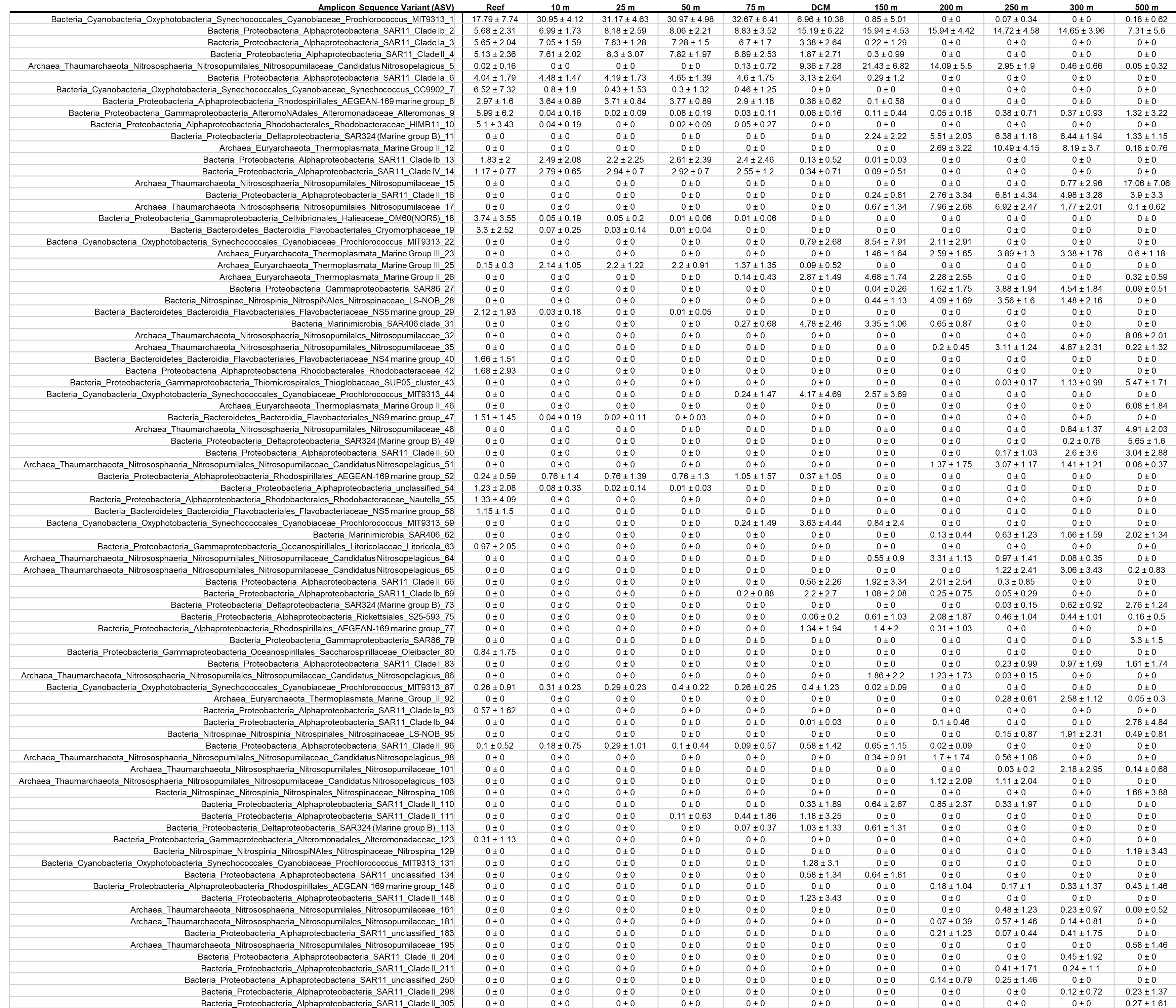

Supplement: Supplementary file 8 — Supplementary Table 1. Mean relative abundances and standard deviations for ASVs with an abundance greater than 4% in three samples, or 8% in one sample. [file EMI-24-4193-s010.tif]

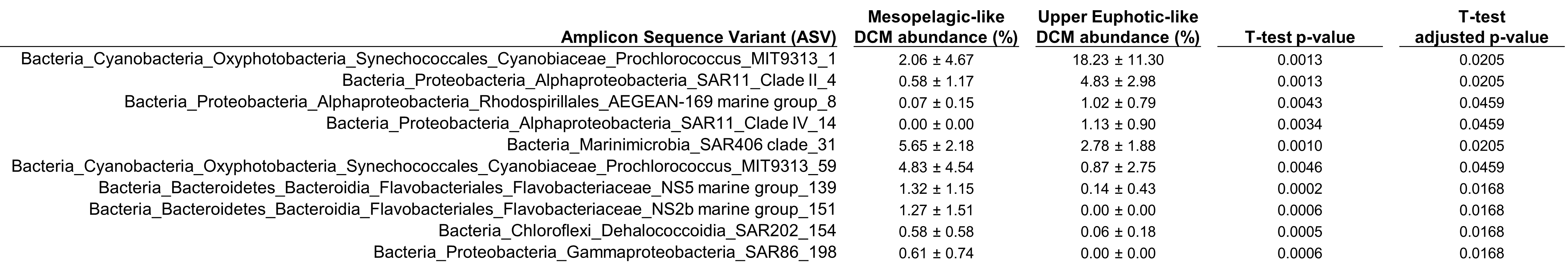

Supplement: Supplementary file 9 — Supplementary Table 2. Mean relative abundances for DCM ASVs enhanced in Mesopelagic‐like and Upper Euphotic‐like stations with T‐test p‐values. [file EMI-24-4193-s004.tif]

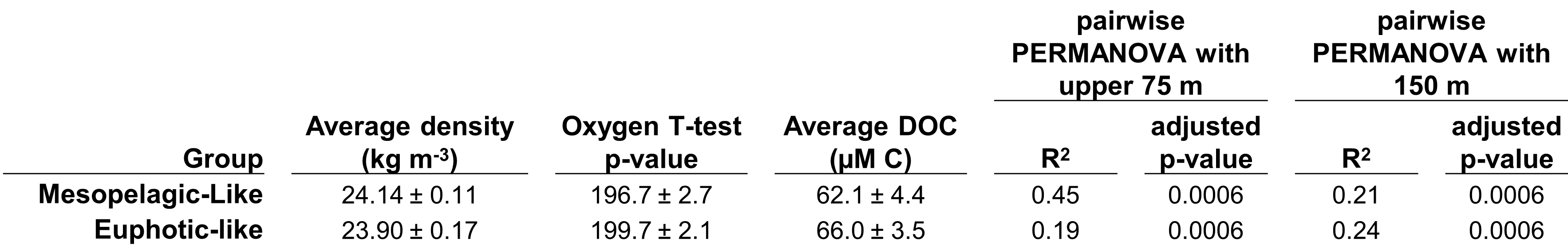

Supplement: Supplementary file 10 — Supplementary Table 3. Mean density, oxygen and DOC concentrations alongside pairwise PERMANOVA R 2 and p‐values for the mesopelagic‐like and upper euphotic‐like DCM groups. [file EMI-24-4193-s002.tif]
